# Kids’ Emotion Recognition Using Various Deep-Learning Models with Explainable AI

**DOI:** 10.3390/s22208066

**Published:** 2022-10-21

**Authors:** Manish Rathod, Chirag Dalvi, Kulveen Kaur, Shruti Patil, Shilpa Gite, Pooja Kamat, Ketan Kotecha, Ajith Abraham, Lubna Abdelkareim Gabralla

**Affiliations:** 1 Symbiosis Centre for Applied Artificial Intelligence (SCAAI), Symbiosis International University (Deemed University), Pune 412115, India; 2 Computer Science and Information Technology Department, Symbiosis Institute of Technology, Symbiosis International University (Deemed University), Pune 412115, India; 3 Machine Intelligence Research Labs (MIR Labs), Auburn, WA 98071, USA; 4 Department of Computer Science and Information Technology, College of Applied, Princess Nourah bint Abdulrahman University, Riyadh 11671, Saudi Arabia

**Keywords:** kids’ emotion recognition, FER, explainable artificial intelligence, LIRIS, children emotion dataset, online learning

## Abstract

Human ideas and sentiments are mirrored in facial expressions. They give the spectator a plethora of social cues, such as the viewer’s focus of attention, intention, motivation, and mood, which can help develop better interactive solutions in online platforms. This could be helpful for children while teaching them, which could help in cultivating a better interactive connect between teachers and students, since there is an increasing trend toward the online education platform due to the COVID-19 pandemic. To solve this, the authors proposed kids’ emotion recognition based on visual cues in this research with a justified reasoning model of explainable AI. The authors used two datasets to work on this problem; the first is the LIRIS Children Spontaneous Facial Expression Video Database, and the second is an author-created novel dataset of emotions displayed by children aged 7 to 10. The authors identified that the LIRIS dataset has achieved only 75% accuracy, and no study has worked further on this dataset in which the authors have achieved the highest accuracy of 89.31% and, in the authors’ dataset, an accuracy of 90.98%. The authors also realized that the face construction of children and adults is different, and the way children show emotions is very different and does not always follow the same way of facial expression for a specific emotion as compared with adults. Hence, the authors used 3D 468 landmark points and created two separate versions of the dataset from the original selected datasets, which are LIRIS-Mesh and Authors-Mesh. In total, all four types of datasets were used, namely LIRIS, the authors’ dataset, LIRIS-Mesh, and Authors-Mesh, and a comparative analysis was performed by using seven different CNN models. The authors not only compared all dataset types used on different CNN models but also explained for every type of CNN used on every specific dataset type how test images are perceived by the deep-learning models by using explainable artificial intelligence (XAI), which helps in localizing features contributing to particular emotions. The authors used three methods of XAI, namely Grad-CAM, Grad-CAM++, and SoftGrad, which help users further establish the appropriate reason for emotion detection by knowing the contribution of its features in it.

## 1. Introduction

### 1.1. Facial Emotions

Facial emotions depicted by humans are naturally understood signals that help humans communicate in a better way. We as human beings feel emotional states and intentions from any video or image showing persons in it. Such a complex set of emotions is evidently easily understood by humans but not by machines. These emotions are depicted, most of the time, visually followed by the use of audio and language. In a recent study [1], according to a psychologist, visual factors contribute to 55% of the emotional understanding; audio contributes 38%, which usually can be categorized according to rhythm, pitch, or tone; and language contributes 7%, which is subject to the language complexity of all the languages around the world.

### 1.2. Need of Facial Emotion Recognition

**Figure 1:**
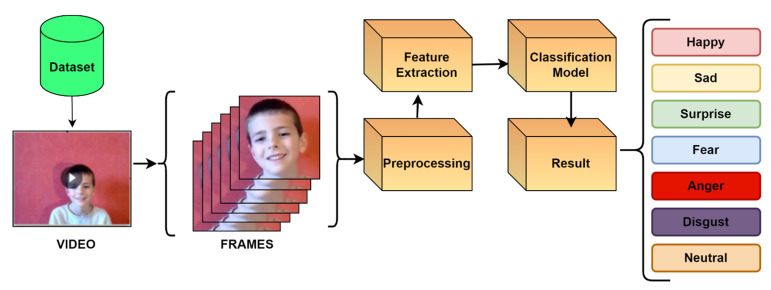
Basic emotion detection using video.

Facial emotions hold the largest emotion-recognizing factor of a human being, and they act as a key factor to understanding to perceive the state of mind of any human being and then to accordingly act. This has become a key reason for important research topics wherein numerous hypotheses, research, and practical research are still being carried out. These studies of human FER are being widely used among many applications such as robotics, cybersecurity, Facebook, Instagram, mental health evaluation, pattern recognition, and other fields according to a study [2]. With FER, one could take advantage of the most natural feedback to digital platforms and hardware technology in the form of emotions, which will help to understand and perceive any product, idea, or platform according to the audience view. These are complex emotions according to a study [1] that discussed about wheel emotion and observed that there are some emotions globally understood without any hindrance. These emotions include happy, sad, surprise, fear, anger, disgust, and neutral. From FER, according to studies, it was found that FER has fine-tuned itself into four layers as evident in Figure 1, where the layers include (1) face detection, (2) preprocessing the data, (3) feature extraction, and (4) emotion classification using models.

From Figure 1, it is evident that, from the frames of the video of children, faces are detected and stored in the first stage, which is also known as the preprocessing stage. These facial components include the eyes, cheeks, eyebrows, mouth, and chin. The second stage will extract more informative features from various face parts such as facial landmark points, face construction, gesture of face, etc. Finally, using the training data, a classifier is trained before being used to generate emotion.

### 1.3. Use of FER in Online Teaching

In times of the current pandemic, the authors have observed that people have been forced to adopt new digital lifestyles, including online teaching. With online teaching, new challenges arose to understanding children’s emotion using explainable factors to gauge and perfect the effectiveness of online teaching, similar to the ones carried out in the adult category in another study [3]. This project aims to develop a method for teachers to understand the emotional state of kids using a normal CNN during online school learning to create activities or study methods to comprehend children’s learning moods as well, which will help teachers understand the reasoning on how such emotions are detected using the explainable artificial intelligence (XAI) method. It aims to identify the real-time emotional state of the end-user and classify it accordingly based on video inputs by training and testing on own created dataset of children between the age group of 7 and 10. Here, a normal CNN was employed, and for every CNN, comparative analysis was provided. The authors’ methodology discussed in this study was to contribute an additional feature to the ongoing online teaching–learning curriculum that will provide an extra edge to the teachers by identifying children’s emotions and giving the teachers an overview of the emotional state of the students according to a recent study [4] by using different XAI techniques, which provide the user explanations that are generated alongside emotion category results that will help to gain teachers’ trust of children. Using XAI, a user can recognize how different features are contributing for each emotion using easily understandable colorful highlighted visualization of different features of the face. Therefore, the authors presented three XAI methods—Grad-CAM, Grad-CAM++, and ScoreCam—in which its features can significantly improve the way of teaching, catering to the needs of the students and improving the overall quality of experience in such dire times.

## 2. Literature Review

Case Study: This case study deals with the topic of online school kids’ emotion detection. In these times of the current pandemic of COVID-19, the authors have observed a forced trend to adopt new digital lifestyles, including online teaching. Most schools and universities have shifted their teaching from offline to online mode or hybrid mode. Though the study material and syllabus remain the same, handling online teaching is still challenging compared with conventional physical classes. Online schools and colleges have two critical stakeholders: digitally migrant teachers and amateurish young kids. Therefore, to understand the teaching effectiveness of students, analysis of their emotions is performed to find their learning moods, which is crucial as it would help both teachers and students become less dependent on physical education. The emotion of students has always been one of the key factors of their attention in studies, whether it is offline or online. For this, finding out the learning mood of students is one of the most critical indicators to gauge the effectiveness of online learning. However, there are no automated tools available that can assist teachers in knowing their students’ emotions and analyzing their learning moods of the ongoing online classes, and in gauging the effectiveness of teachers’ teaching and subject understanding of students. To understand more about the work carried out in this field, the authors performed a literature review on the same, which is indicated in Table 1.

The authors of [5] have suggested an online tutoring system also known as Remote Lab (RL). This module uses clmtrackr, a JavaScript library, to detect and extract facial coordinates. Then a machine-learning algorithm is used to identify the expressions. However, another study [6] also emphasized other components to study FER by introducing the student emotion recognition system (SERS) concept, where the movement of eyes and the rotation of the head are also used. The Viola Jones algorithm and LBP have been used for face detection and feature extraction. The SERS has been implemented in MATLAB with three concentration levels: high, medium, and low. Now, although FER may be divided into audio and video components, to understand it, more merging of both these components is absolutely necessary; this has been shown by a study [7] where the authors proposed the vocal emotion recognition part of the FILTWAM (Framework for Improving Learning Through Webcams and Microphones) framework. For extraction, they used sentence-level segmentation, and for classification, the minimal sequential optimization (SMO3) classifier of WEKA software, a software tool for data mining, was used. The results obtained from this study were taken forward by another study [8], which fused the outputs obtained from the visuals from a web camera and audio from a microphone, which shows how multimodal models perform in emotion recognition. Now, although the basic methods for emotion classification may remain the same, the set of approaches taken for a particular dataset is always different, which often leads to different variants of the results. Such an example can be withdrawn from a study [9] where OpenCV was used to detect faces, and for classification, they used a deep CNN with SGD. Meanwhile, another study [10] used haar cascades for face detection and predicting emotion using the eyes and mouth section, and the novel Sobel edge detection eyes method was also used for classification. Furthermore, another study [11] used Praat 6.0.36 for feature extraction, and data-preprocessing SVM with RBF kernel was used. For offline classification, SVM was used, and for online classification, the models were trained in WEKA.

Now, although there have been different implementations carried out on kids’ emotion dataset, it was also observed that there is a scarcity of such dataset as there are not as many datasets of kids as compared with adults, which is indicated in a latest study [1]. It is also observed that, although the datasets of kids’ emotion are few, the individual quantity is less but the emotions recorded are also different as shown in Table 2. This table also shows the parameters of every dataset including the author-created novel dataset, which has covered seven emotions shot during online school classes at the time of the COVID-19 pandemic. Now, as per Table 2, most of these datasets only have videos or images; however, only one dataset includes the visual and audio cues, which is EmoReact. It is also observed that, compared with the adult datasets, the size of the datasets is very small, except for the LIRIS and EmoReact datasets, which contain 208 and 1102 videos, respectively. The rest of the datasets contain a few hundreds of pictures of emotional data, which can be used for testing purposes for transfer learning. Most of these datasets have different emotions displayed, and not all have the same number of emotions displayed. Interestingly, from Table 2, the authors observed that the subjects in these datasets are above 10 years of age, which made them decide to create their own dataset where the subjects are below 10 years of age, showing various emotions. Compared with other datasets, the authors’ dataset was shot in a different environment. Here, DEFSS [12], NIMH-Chefs [13], and CAFÉ [14] were shot in labs, whereas only EmoReact [15] has a dataset shot from online web sources showing simultaneous emotions. LIRIS has also shot simultaneous emotions but at lab and home only. The authors used LIRIS [16] in the proposed study, and based on the LIRIS dataset environment, they also created their own dataset at lab and home with simultaneous and posed emotions. Moreover, from these datasets in Table 2, the authors have chosen the LIRIS dataset as part of their study, along with their dataset in this paper. It was also observed that the LIRIS dataset paper [16] has so far achieved 75% accuracy using VGG, which is a deep-learning model, and no other study has worked further on this.

## 3. Motivation

After an exhaustive literature review, it was observed that there had been significantly less work carried out in the field of kids’ emotion detection. There is no available dataset showing the emotions of children below the age of 10. In addition, there has been no progress in the accuracy achieved in all these kids’ datasets, as none of them crosses the benchmark of more than 75%. Moreover, there has been no comparative analysis of CNN implementation on these datasets such as LIRIS and the self-created dataset, which will be a great addition in the field of FER. This has motivated the authors and has become the main essence of the topic, which is further covered in the following sections.

## 4. Contribution of the Paper

- This study significantly contributed to an increase in the accuracy of LIRIS as compared with the created dataset’s accuracy, which no other study in the field of FER obtained.
- As per the literature, it was observed that there is a lack of emotion datasets from kids of ages 7 to 10; therefore, the authors proposed their dataset with kids from this age group.
- The authors used seven different CNNs for classification and performed a comparative analysis on both the LIRIS and author-created datasets.
- The study focused on how children’s face is different from that of adults according to a recent study [17], and to understand more about the emotion, the authors used seven different CNN classifications using 3D 468 landmark points and carried out a comparative analysis of both the LIRIS and authors’ proposed datasets.
- This study also focused on how these seven different CNNs recognize emotions on the dataset using XAI, namely using Grad-CAM, Grad-CAM++ and SoftGrad.

## 5. Proposed Model

**Figure 2:**
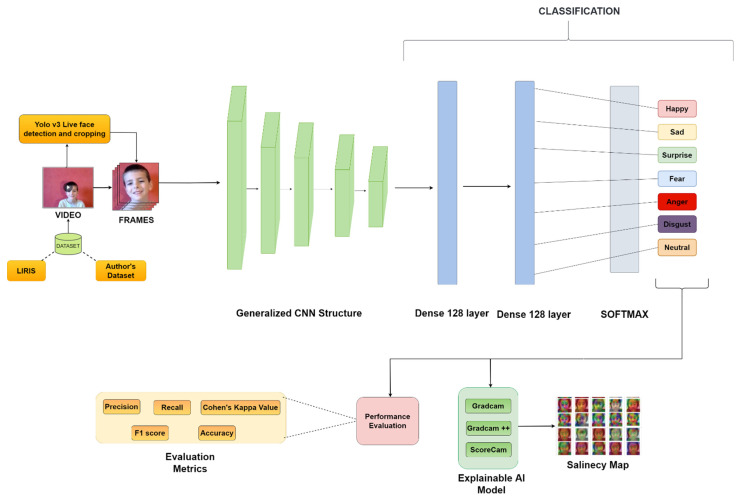
Architecture diagram of proposed emotion recognition.

After the literature review, the authors observed a scarcity in kids’ emotion datasets and saw that most of these datasets have not even achieved more than 75% accuracy after applying various approaches based on a latest study [1]. To challenge this, the authors created their own dataset and used the LIRIS dataset as well, and they also created separate versions of the datasets called LIRIS-Mesh and Authors-Mesh by using 3D landmark points, which were later experimented with various deep CNN models; the authors performed a comparative analysis of all the dataset types using the wide array of CNNs used. The authors also depicted their approach in the form of a diagram in Figure 2, where both LIRIS and authors’ datasets simultaneously got preprocessed using YOLOv3, then a generalized CNN was applied on them, layers were added further to make them effective, and then a wide array of performance metrics was accordingly used for each CNN on the dataset. Along with that, the authors also used XAI methods to explain how each model for every dataset type perceives the emotions displayed by the model used.

## 6. Breakdown of Proposed Emotion Recognition in Brief

### 6.1. Dataset

#### 6.1.1. LIRIS Dataset

**Figure 3:**
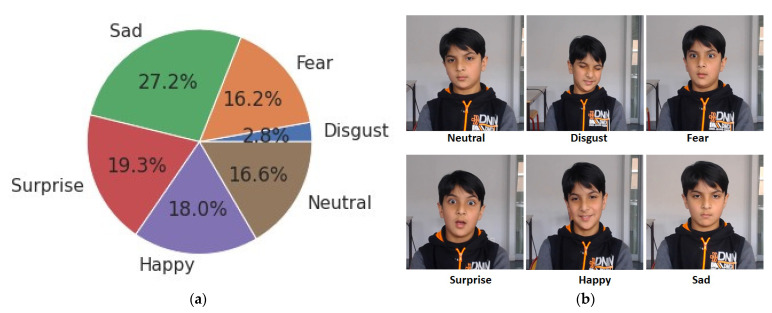
(**a**) LIRIS emotion distribution. (**b**) Publicly allowed sample images of LIRIS emotions.

The LIRIS dataset contains five universal expressions except for the neutral emotion. The dataset was recorded using a webcam, and the expressions were spontaneous. This dataset contains 206 video clips of 12 subjects with 24,000 emotional frames with a 30 fps rate. The authors trimmed the starting and ending parts of the videos not present in the original dataset to create a neutral category. This helped the authors to increase the performance since they removed the bits from the front and end of the video, and now the AI model will only focus on getting trained on the emotion-displaying section rather than the non-emotion-displaying section such as neutral, which acts as a noise in training. Now, once the neutral emotion is included in the study, the total count of emotions is now six. The authors observed that the dataset was not balanced, as shown in Figure 3a; this was because the video duration of each category of emotion differs video by video, which impacts on a number of extracted training and testing video frames, resulting in imbalancing. Furthermore, it should be noted that the “anger” emotion is excluded, as the number of videos in the anger category is very few to even train. As observed from Figure 3a below, there are fewer video frames for the "disgust" emotion and more video frames for the "sad" emotion because of the video duration of the emotion. However, the image quality is very good as per Figure 3b.

#### 6.1.2. Author Dataset

**Figure 4:**
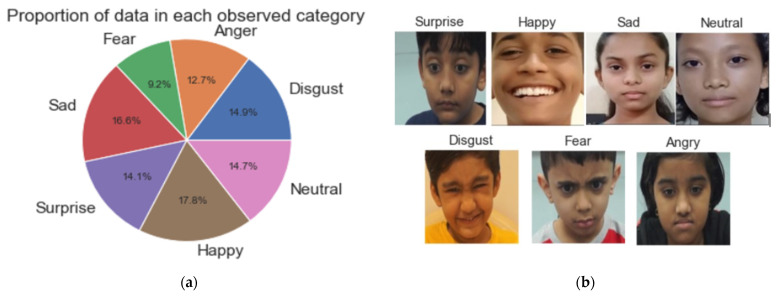
(**a**) Authors’ emotion database distribution. (**b**) Sample video frame images captured from authors’ emotions database.

Based on the review, it was evident that there are fewer kids’ emotion datasets; to tackle this situation, the authors created their own dataset made out of children belonging to the following nationalities: India, Bangladesh, and Nepal. India is a diverse country, which represents multiple communities, ethnic groups, religions, and races. The dataset holds most children from India because, for the authors, Indian children were more easily accessible than children from other countries, which resulted in few inclusions of children from other countries. All subjects in this dataset are strictly under the age range of 7–10, consisting of children from all races, religions, communities, and geographical backgrounds. Now, as compared with LIRIS, the authors tried to create equal duration of videos as much as possible, which will help to balance the data during training and testing; however, as some emotions can last only a few seconds, it makes it harder to train on that category because of dataset bias introduced due to the unavoidable variable length of the recorded videos of children, as these children are not child actors and it is not easy to bring up emotions on them on a whim; moreover, the duration of facial emotion expression differs from subject to subject. To record this dataset, instead of using a webcam to record or take videos from movies or TV clips, the authors used a professional setup with great facial lighting conditions shot in a peaceful environment without any external elements interfering in the creation of the dataset. The authors used a DSLR professional camera that can record high-definition videos at 60 frames per second. This allowed the authors to record more details at a better resolution. The authors’ dataset contains 81 videos of 12 subjects with 12,000 emotional frames. A higher framerate allowed authors to extract more frames when preprocessing. This dataset is more diverse since the authors used a better subject-to-video ratio. The children were instructed to pose for the emotions, spanning 4 to 8 s per video. Half of the dataset consists of 81 posed videos whose emotion distribution can be seen in Figure 4a, and its sample image can be seen in Figure 4b. The other half consists of eight online study lectures recorded during online school at the time of the COVID-19 pandemic. Each recorded video spanned from 10 to 15 min, which also included the spontaneous expressions of the children. This half of the dataset can be used to test the real-life scenario of online lectures, which is not found anywhere in the field of FER.

### 6.2. Preprocessing for Visual Features

For emotion detection from videos, the videos need to be preprocessed into thousands of frames of emotional data. After this, face detection is used, and the detected faces are then later cropped, which will help the researcher give the best results. To achieve these three things, the authors used the YOLOv3 face detection model, which is available online.

#### 6.2.1. Face Detection

**Figure 5:**
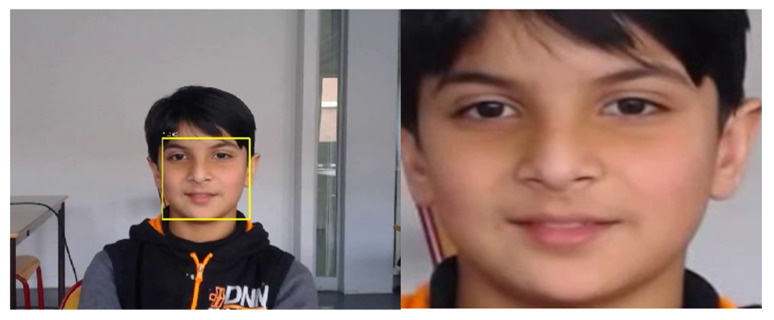
YOLOv3 simultaneous face detection and cropping.

The first step in any facial emotion detection system is to detect a face. To detect faces, the authors initially have decided to use the OpenCV face detection algorithm on the LIRIS dataset, which is full of short videos. Still, the problem was that it was showing a data loss, and the amount of time required for detection was high. Such an application would have been useless in the real world if it takes more time to detect the face. The authors decided to use the YOLOv3 [18] face detection model in a darknet neural network pretrained on the WIDER FACE dataset using online and readily available sources to tackle this problem by referring to another study [18]. This made the model detect faces on the directories of videos at a much faster rate and with good precision, as seen in Figure 5.

#### 6.2.2. Face Cropping

In video emotion classification, videos are not directly used; they are first converted into video frames, which are nothing but a series of images one after the other displayed in a particular timestamp that needs to be classified. The video classification problem has now become an image recognition problem. To solve this problem, whatever face is detected using the YOLOv3 model, that face’s dimensions are noted against the frames of those videos and are resized into a 224 × 224 size, cropped using the OpenCV face cropping feature, and then stored in a temporary directory. The sample of a cropped image can be seen in Figure 5.

#### 6.2.3. Face 3D Landmark Generation

**Figure 6:**
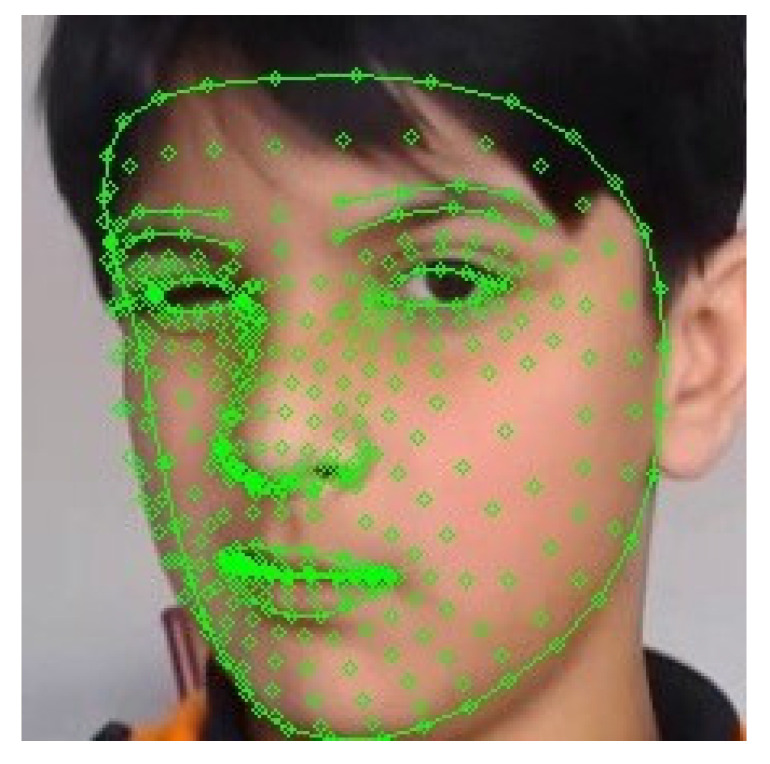
Face mesh/468 landmark points on disgust emotion.

Usually, when a human sees an averted emotional face, it almost gives 100% accuracy on the kind of emotions expressed. The human sees a face as a 3D object and has gained sufficient experience from the human community. When a face is averted, geometric features such as 72 landmark points cannot be observed, hence making it difficult for AI to understand. To tackle this, the authors implemented Google’s famous MediaPipe Face Mesh generation algorithm, also previously used in FER in a recent study [19], which creates 468 landmark points of the images, similar to a 3D face model, which is far more advantageous than a 2D landmark according to a study [20]. These face mesh images are then stored in another temporary file, which can be used later as additional training dataset components, namely LIRIS-Mesh and Authors-Mesh. It is to be noted that a separate testing video was used that was not used in the training. The sample image for 3D 468 landmark points can be seen in Figure 6.

#### 6.2.4. Data Augmentation

For augmentation, the authors used the TensorFlow imagedatagen library using the processing input unit of the EfficientNet network [21]. This was chosen because the architecture in itself uses a scaling method using a compound coefficient on an image width and resolution. Like most of the augmentation conventional methods arbitrarily scaled, EfficientNet uses uniform scaling such that whenever the image has more resolution or width, then the EfficientNet as additional layers and channels will be added to the augmentation process unit to expand the receptive field and capture more fine-grained patterns on a bigger image. Since the video frame quality is good enough but shot in a different background environment and lighting in both the LIRIS and authors’ datasets, the EfficientNet preprocessing network turns out to be the best fit for further augmentation.

### 6.3. Deep Models Used

**Figure 7:**
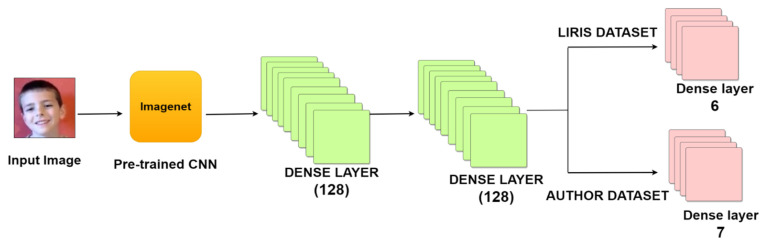
Proposed CNN structure.

The authors used convolutional neural network methods for facial emotion recognition. They are easy to train and give better results than most methods. However, the results obtained are deemed worthy of training time, as the CNN records the edge and corner of an image, which is a basic feature. Then complex features such as textures or shapes are captured by the next layer, and then all the remaining upper layers perform the same task in order to learn complex emotions. Now, if the CNN is trained from scratch, the weight of the training is set according to the dataset distribution, which is not favorable in an imbalanced dataset. Hence, the authors used a balanced weight that was calculated as per the dataset distribution for fair classification without any bias, which acts a means for fair unbiased training. Now, as there are multiple CNN models and different CNN models have different architectures, to maintain a general flow between all the selected models for classification, the same set of additional layers, which are 128 dense layers, was added, and then another set of 128 dense layers was added for fine-tuning without hindering any single CNN while training. However, it is to be noted that the authors used readily available TensorFlow’s pretrained CNN models for FER. Then for the LIRIS dataset, as there are six emotions, dense six layers were, therefore, added, and similarly, dense seven layers were added for the authors’ dataset, which is clearly shown in Figure 7, describing the proposed facial emotion recognition CNN method in detail with required illustrations. There were seven pretrained mCNN models used for comparative analysis, which is covered in further subsections.

#### 6.3.1. VGGNets

With 16 convolution layers and 3 completely connected layers, VGG19 consists of 19 layers. It is a deep CNN that is mostly used to recognize face emotions. The network only employs 3 × 3 convolutions, which gives it more depth according to a study [22]. This model was used to classify picture objects using the LIRIS and authors’ datasets. Therefore, to recognize a facial image as one of the seven emotion classes mentioned above, we substituted the last dense layers of the pretrained model with the new dense layer(s). The architecture was then fine-tuned using the convolution basis of the pretrained model as well as the extra dense layer(s). This cleaned dataset was then cropped and resized through preprocessing for fine-tuning, where the last layer of the pretrained network was removed and replaced with relevant new layers. Now, for the training, triplet loss function was used; that is, for the training, a model requires three images—the anchor, positive, and negative images—which are then flattened, and then the convolution is formed on it by VGGNet. These images are then fed into the VGGNet layers from where the emotions are recognized.

#### 6.3.2. ResNets

The main problem with VGG networks was the number of layers. As the number of layers increased, the network became deeper, and, hence, it was difficult to train, resulting in the degradation of the accuracy. To tackle this problem, ResNets were introduced [23]. Multiple layers are added to the networks to solve complex problems, which triggers the vanishing gradient problem. However, ResNets have residual blocks. A direct connection is used in a residual block to skip some layers in between. It is called skip connections. This helps the network tackle the vanishing gradient problem since these connections allow the gradient to flow through a shortcut path. It allows the higher layers to perform as well as the lower layers. In our module, the authors used three different versions of ResNets: Resnet50V2, Resnet101V2, and Resnet152V2. Normally in ResNet, first, the image is flattened into column vectors and given back to the feed-forward neural network, converted into a format suited for multilevel perceptron. Each training cycle is then applied to the flattened data. As a result, the model can distinguish between the image’s key characteristics and some low-level features. ResNet next performed convolution on the input, followed by four residual blocks, and lastly, full connection, which emerges at the end of the CNN to summarize the features of the preceding layers’ operations and execute classification tasks.

#### 6.3.3. DenseNets

The DenseNet models used are DenseNet121, DenseNet169, and DenseNet201, with 121, 169, and 201 layers, respectively. Each layer in the dense convolutional network (DenseNet) design is directly connected to the next feed-forward [24]. Meanwhile, the L layer of a typical convolutional neural network (CNN) has an L relationship, where the relationship with each other is L (L + 1)/2. DenseNet has an extremely narrow layer (12 filters per layer) with a tiny collection of feature mappings in the aggregate information of the network. DenseNet has the following advantages: it is light on the gradient problem, feature deployment, and feature reuse; and its functionality minimizes the number of parameters. The authors from study [25] found that DenseNet-201 is a convolutional neural network with 201 layers. Batch normalization (BN), ReLu activation, and convolution with a filter are all included in each layer. In DenseNet architecture, each block has an image pixel input in the form of a matrix, which is subsequently passed to the batch normalization stage, which helps reduce overfitting during training. If the value is negative, ReLu activation will convert it to positive, but it will not change if the value is not smaller. To process a matrix picture that has passed the ReLu activation step, a convolution matrix with a filter will be multiplied by a matrix. The resulting output will be a previously processed matrix value.

#### 6.3.4. InceptionV3

The authors used InceptionV3 because of the well-known "inception layers" in this model, which overcome the challenges of a deeper network [26]. Basically, this model uses multiple-size filters on the same layer, making the network larger rather than deeper. The inception module is what you are looking for. The inception layer comprises 11 convolutional layers, each of which has its output filter banks concatenated into a single output vector that serves as the input to the following stage where several filters improve accuracy while decreasing computing complexity. To avoid overfitting, factorized convolutions, batch normalization, RMSprop optimizer, asymmetric convolutions, smaller convolutions, and label smoothing were introduced. This model is fed with augmented images during training.

#### 6.3.5. InceptionResNetV2

In InceptionResNet, the inception architecture is combined with residual connections called the inception-ResNet block. Different-sized convolutional filters are combined with the residual connections in this block. The degradation problem is solved because of the residual connections, and it drastically reduces the training time. Because of the inception modules, the network is more comprehensive than is deeper. The V2 of the InceptionResNet model has the same training time as Inception v4. In this architecture [27], the inception blocks are less computationally expensive than the original inception blocks utilized in Inception V4. Now, as per its architecture, each inception block is followed by a filter expansion, which consists of 11 convolutional neural networks without activation. This increases the filter bank’s dimensionality to match the input depth to the following layer; also, residual connections are used to replace the pooling procedure inside the inception blocks; furthermore, pooling procedures, on the other hand, are usually seen in the reduction blocks of this CNN. Moreover, this CNN does not employ batch normalization, which is also explained in a recent study [27]. This is performed to make the model smaller and trained on a single GPU. The image is supplied to these blocks in vector form, and emotions are formed.

### 6.4. Explainable AI

**Figure 8:**
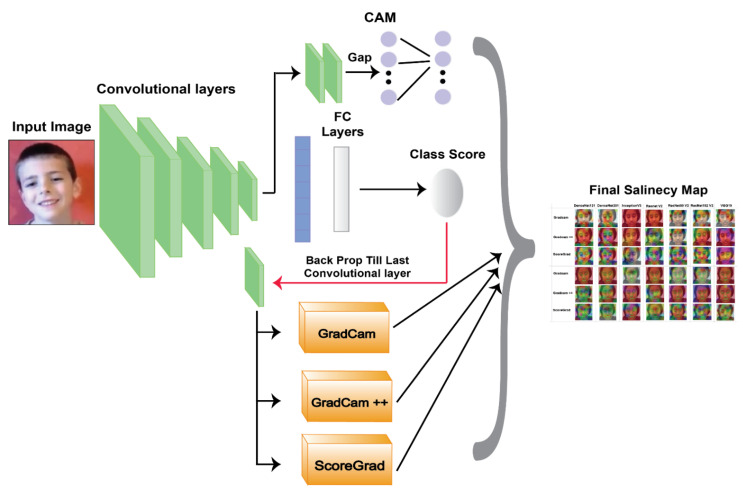
Explainable AI implementation.

With the deep-learning model, the authors successfully carried out the user’s emotional recognition, but to gain the user’s trust, it is essential to explain how the input features of a machine-learning model affect its predictions. Hence, the authors can explain to the users the question of "Why?", that is, why the emotion is sad, happy, and so on. When users find features that contribute to each emotion, they can understand each emotion’s reason. Hence, to achieve this, the authors applied three different XAI methods: (1) Grad-CAM, (2) Grad-CAM++, and (3) ScoreGrad, which are shown in Figure 8 and explained in further subsections.

#### 6.4.1. Grad-CAM

Grad-CAM uses the gradient information coming into the last convolutional layer of the CNN to assign priority values to each neuron for a particular choice. For any class *c*, obtain the class-discriminative localization map Grad-CAM

(1) $$L_{Grad-CAM}^{C}\in {\mathbb{R}}^{u\;X\;v}$$

of width *u* and height *v*. The authors of [28] initially computed the gradient of the score for class *c*, *y^c^* for feature map activation *A^k^* of a convolutional layer (before the softmax), i.e.,

(2) $$\frac{\partial y^{c}}{\partial A^{k}}$$

These gradients are global-average-pooled twice over the width and height dimensions to obtain the neuron significance weights (indexed by *i* and *j*, respectively).

(3) $$a_{k}^{c}=\frac{1}{z}\;\sum_{i}\sum_{j}\frac{\partial y^{c}}{\partial A_{ij}^{k}}$$

When back-propagating gradients to activations and computing $a_{k}^{c}$, the actual amount of computation of consecutive matrix products of the weight matrices and the gradient to activation functions until the final convolution layer in which the gradients are propagated. As a result, this weight ack is a partial linearization of the deep network downstream from *A*, capturing the "importance" of feature map *k* for a target class *c*.

The authors performed a weighted combination of forwarding activation maps and followed it by a ReLu to obtain

(4) $$\;L_{Grad-CAM}^{C}=ReLu\left(\sum_{k}a_{k}^{c}A^{k}\right)$$

Because the authors were only interested in elements that have a greater impact on the image’s emotion, such as pixels whose intensity should be enhanced, they used ReLu on the linear combination of maps to enhance *y^c^*. Brightening pixels in an image with fewer contributing or influencing elements is more likely. As one may assume, localization maps without this ReLu sometimes reveal darker parts than the targeted emotion-recognizing features, and the end results obtained using Grad-CAM are similar to those of the ones carried out in a recent study [28].

#### 6.4.2. Grad-CAM++

When an image has many occurrences of the same class, Grad-CAM fails to appropriately locate the objects. Multiple instances of the same object in an image are prevalent in the actual world, so this is a serious problem. Another consequence of utilizing an unweighted average of partial derivatives is that the localization does not necessarily correspond to the entire item but rather to parts of it. Grad-notion CAM of making a deep CNN more transparent could be jeopardized as a result of this. Hence, the authors of [29] used a Grad-CAM plus model, which is created by directly describing the impacts of each pixel in a CNN’s feature maps to the final result. In particular, Equation (1) has been reformulated by directly coding the composition of the weights $w_{k}^{c}$ by the authors:

(5) $$w_{k}^{c}=\sum_{i}\sum_{j}a_{ij}^{kc}.relu\left(\frac{\partial Y^{c}}{\partial A_{ij}^{k}}\right)$$

where relu is the rectified linear unit activation function. The notion is that $w_{k}^{c}$ represents the significance of a specific activation map $A^{k}$. Positive gradients have been demonstrated to be important in constructing saliency maps for a given convolutional layer in previous efforts in pixel-space visualization such as deconvolution and guided back-propagation. A positive gradient at the location (i,j) for an activation map $A^{k}$ implies that increasing the intensity of the pixel (i,j) would have a positive influence on the class score $Y^{c}$. A linear combination of the positive partial derivatives with respect to each pixel in an activation map is thus obtained and elucidates the significance of that map for class c. The weights $w_{k}^{c}$ are a weighted average of the gradients instead of a global average, thanks to this structure.

#### 6.4.3. ScoreGrad

Variations of Grad-CAM, such as Grad-CAM++, only differentiate in combinations of gradients to represent $a_{k}^{c}$. They seek to generalize models that do not have global pooling layers, so they can be widely used. ScoreCam is a post hoc visual explanation method, and the value of activation maps is encoded by the global contribution, which bridges the gap between perturbation-based and CAM-based techniques of the corresponding input features rather than the local sensitivity measurement, also known as gradient information, which represents the weight of activation maps in an intuitively understandable way.

In contrast to earlier techniques [30], the authors added the importance of increasing confidence into the gradient information going into the last convolutional layer to indicate each activation map’s relevance. Taking a convolutional layer into account, l ScoreCam will be

(6) $$L_{Score-CAM}^{C}=ReLu\left(\sum_{k}a_{k}^{c}A_{l}^{k}\right)$$

## 7. Experimental Results

### 7.1. Results of Deep-Learning Models

The authors evaluated three kinds of performance metrics: precision, recall, and F1 score. These metrics are calculated with the help of AI. The equations for precision, recall, and F1 score are given in (7)–(9) as follows:

(7) $$Precision=\frac{True\;Positive\;}{True\;Positive+False\;Positive}$$

(8) $$Recall=\frac{True\;Positive\;}{True\;Positive+False\;Negative}\;$$

(9) $$F1\;Score=\frac{True\;Positive\;}{True\;Positive+False\;Positive}\;$$

### 7.2. Explaining Explainable AI Visualizations

**Figure 9:**
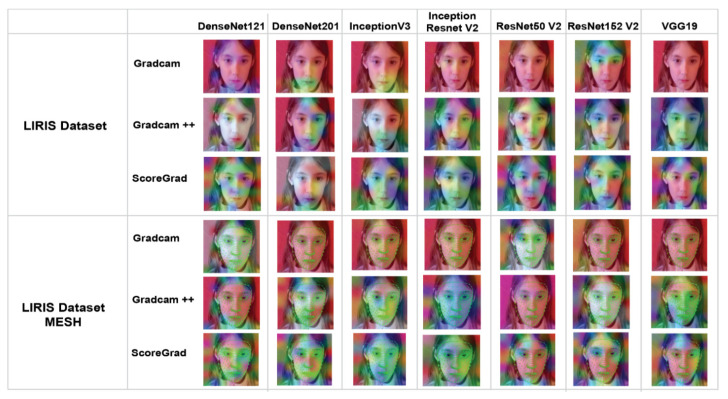
Saliency map of LIRIS dataset.

**Figure 10:**
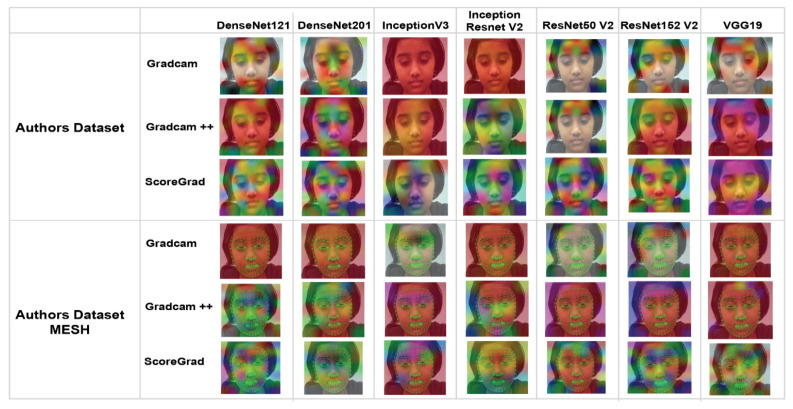
Saliency map of authors’ dataset.

Explainable AI not only creates heatmap but also helps to visualize emotions better through variations of colors. The authors applied three explainable AI methods, the output of which is a heatmap visualization for a given class label: (1) Grad-CAM, (2) Grad-CAM++, and (3) ScoreGrad. The authors applied all these methods to the LIRIS and authors’ datasets and created a saliency map of all CNN models similar to the one in a recent study [31]. Figure 9 shows the saliency map of the LIRIS dataset, and Figure 10 shows the saliency map of the authors’ dataset for all three explainable AI methods on different CNN models.

**Figure 11:**
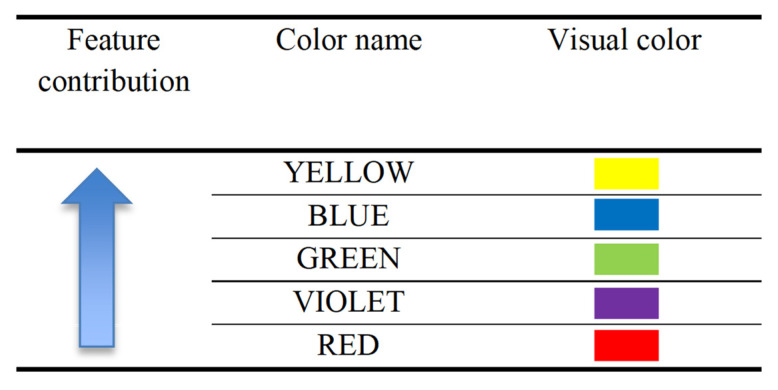
Feature contributing colors.

Grad-CAM visualizations of the model, as shown in Figure 9, divulge that the model not only has learned to predict the emotion but also presented feature contribution to that prediction. In Figure 9 and Figure 10, as we horizontally move, we can see the explainable visualizations on different models used to detect the emotions, and as we vertically move, we see different explainable models used to explain the predictions. Hence, from the saliency map, which is an explanation method used to interpret predictions shown in Figure 9 and Figure 10, we can infer that ScoreCam gives us clearer visualizations and Grad-CAM gives the least, giving us ScoreCam as the final model used to explain the features contributing in detecting the emotions. A saliency map of each image shows part of the image contributing the most to the activity based on the colors shown in Figure 11.

In Figure 9 and Figure 10, we show two saliency maps, one with a dataset and another with a mesh dataset, where mesh represents features contributing to the facial recognition in a more better way with ScoreGrad giving us the best model to explain which feature is contributing to the model. As we can see in the InceptionResNet V2 model–authors’ dataset mesh–ScoreGrad, that yellow color, as per Figure 11, gives the eyes as the maximum contributing feature in determining the facial emotion. In this way, this saliency map helps us in determining not only the features contributing but also the best model explaining the visualizations of facial emotion recognition such as ScoreGrad.

## 8. Experimental Results

The authors used various deep-learning models on different dataset versions, namely LIRIS, LIRIS-Mesh, authors’ dataset, and Authors-Mesh. In these datasets, the authors ran various models onto them and achieved accuracy above 80% in all dataset versions. This can be observed from Table 3 and Table 4, which show the accuracy obtained by using the LIRIS and authors’ datasets, in which ResNet 152 V2 has performed the highest in both of them, where LIRIS achieved an accuracy of 89.31% and authors’ dataset 90.98%. However, despite the previous work performed in a study that obtained 75% accuracy using VGG, the authors on that very dataset observed 81% accuracy using VGG19 compared with their own dataset in VGG19, in which an accuracy of 84.57% was achieved. In Table 3, the lowest-achieving accuracy was VGG19 itself. The authors realized that the dataset is imbalanced in terms of images extracted from videos because of the variable video size; however, the accuracy metrics are deemed worthy because additional weights were added as per the quantity of each label of emotions in the dataset enabling the model to train in an unbiased way. In addition, in Table 3, it is quite clear that from the standard emotion dataset, it has categorically improved to 80% and above. However, since the dataset was imbalanced, other metrics such as precision, recall, F1 score, and CAPA values hold higher importance over accuracy, as the authors showed in Table 4 and Table 5, which deal with performance metrics concerning the LIRIS and authors’ datasets. These tables show that the F1 score, an essential metric when dealing with an imbalanced dataset, has shown that ResNet 152 V2 has performed the highest in LIRIS, which is 0.8986 out of 1. The lowest was VGG19, with an F1 score of 0.8066 out of 1 in LIRIS, as shown in Table 4. In terms of the authors’ dataset, as shown in Table 5, the F1 score was the highest in ResNet 152 V2, which was 0.9086 out of 1, and the lowest was achieved in VGG19 with an F1 score of 0.8455 out of 1.

Now, to elaborate on how these results were obtained, the authors presented the CNN model details in Table 6, where the pretrained ImageNet CNN models were used and additional layers of 128 units were added twice with a ReLu activation layer, and then the layers were densed into the categorical number of unique emotions present in a database, namely six for LIRIS, which has happiness, sad, disgust, neutral, surprise, and fear. For the authors’ dataset, the same emotions are created with an extra emotion of anger, making it an honest seven emotions in total. Very few anger emotion videos existed in the LIRIS database; however, since the dataset was already imbalanced in terms of variable extracted emotion frames because of variable video durations, this contributed to excluding the anger emotion from the study, making it less even to train; hence, anger emotion from LIRIS was not included in the study. Although the number of emotions was different in both datasets, the model configuration run was the same. To understand more about the model, the authors presented several epochs and batches used for both datasets in Table 7. The LIRIS and LIRIS-Mesh datasets used 30 epochs with a batch size of 32 for every deep-learning model. For the authors’ dataset and authors’ mesh, a dataset of six epochs was used for a batch size of 16. The authors’ dataset used a batch size of 32 for LIRIS because LIRIS has a high quantity of datasets compared with the authors’ dataset. Since the dataset is recorded in different backgrounds and conditions, the noise must be reduced. At the same time, training and the gradient estimates are better on every epoch; however, the time for training required is vast for every CNN, which is about 30 min. Nevertheless, for the authors’ dataset, the batch size selected was 16. This is because the dataset’s size is low, and the lower the batch size, the lesser the regularizing effect, and the lower the generalization error. Also, the lower the batch size, the better it will perform in terms of the computation power required, and the lesser the time to train with an almost similar range of accuracy achieved, which is above 80%, the same as that in LIRIS with a difference in strategic batch size change to prioritize higher accuracy in less time. The authors tested this CNN using different testing datasets on which a model was never trained to check the generalization for both LIRIS and authors’ datasets. Out of the 12 subjects in both datasets, 8 subjects were used for training, 2 for model validation during training to accordingly fine-tune the model, and 2 for separate testing.

Using the same hyperparameter configuration of the model shown in Table 6 and Table 7, the authors created Table 8 that shows the overall accuracy of all four versions of the dataset used. Now, the authors identified that many studies have used only 68 landmark points; however, these were all carried out only on adult datasets, and the landmark points of children and adults may be the same, in general; still, since children’s faces are small and different from adults’, it was deemed that 468-landmark-point datasets are required to cover these identified gaps. Therefore, the authors introduced Table 8, where it is evident that, although the model used was the same, it was observed that the 468-landmark-point dataset has less accuracy, and in terms of the highest accuracy achieved in LIRIS-Mesh, it was ResNet 50 V2, which is about 83.6%, whereas in authors’ dataset mesh, it was 90.87% in ResNet 152 V2.

Finally, to give users detailed explanations of different features to recognize the emotions, the authors provided a heatmap where they can visually understand why a particular image recognizes such emotion. The heatmap shown in the images gives the colors that show the extent of contribution of each feature. In Figure 11, the authors have shown an increasing order of feature-contributing colors. Red gives the minor contribution, while yellow gives the highest contribution.

**Figure 12:**
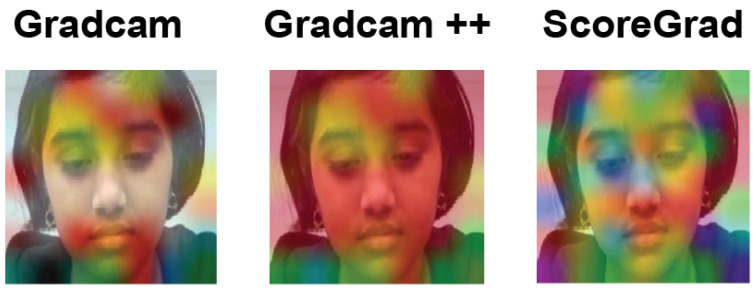
Explained visualization of authors’ dataset.

Therefore, using Figure 11, the authors explained the reasoning for emotion recognition and features contributing to emotions. This makes us understand the heatmap visualization by explaining one sad image from the authors’ dataset passed through the DenseNet121 model. Figure 12 shows the sad emotion image of the DenseNet model, where all explainable AI results are shown. As the authors can see, in Figure 12, heatmap colors become more visible, implying that ScoreGrad has the highest accuracy following Grad-CAM++ and Grad-CAM. Here, the colors are highlighted on the lips as well as the eyes and eyebrows, which become brighter in ScoreGrad, which indicates that these features are providing a maximum contribution toward the sad emotion; changes in these features give us the sad emotion. For example, eyes and eyebrows indicating the blue color in ScoreGrad explain that changes in these features help in recognizing the sad emotion the most as compared with the lips, which is in red color, giving us the least indication of emotion. Hence, we get to know by considering the effectiveness of colors in Figure 11 that changes in which feature determine the emotion the most. In this way, this helps not only in recognizing the emotion but also in understanding the contribution of each feature in the emotion.

## 9. Conclusions

The authors used deep-learning models for children’s emotion detection in online learning platforms on LIRIS and self-made datasets. From the literature, the authors observed a lack of children’s emotional database, so they successfully created a children’s database for kids aged 7 to 10. The literature also observed that adults’ face is different from that of children. The emotions depicted by children on their faces are different, which is why the authors used 3D 468 landmark geometric points of both LIRIS and authors’ generated datasets, creating in total four dataset versions, namely LIRIS, LIRIS-Mesh (3D 468 geometric landmark), authors’ dataset, and Authors-Mesh (3D 468 geometric landmark). The authors used seven different CNNs and achieved significant accuracy, with the highest accuracy among four datasets being 90.98% via an exhaustive comparative analysis. The authors employed three explainable AI algorithms, namely Grad-CAM, Grad-CAM++, and ScoreGrad, to assist users in determining an appropriate rationale for emotion detection by understanding the contribution of its attributes, which will be a great contribution in the field of FER especially in online school learning, which will enhance the learning pace of children on par with offline learning.

## Figures and Tables

**Table 1:** Comparison of different studies carried out in FER.

Reference	Methodology	Modality Used	Issues	Accuracy
[5]	They have used a JavaScript library clmtrackr to detect coordinates of the face and then apply a machine-learning algorithm to identify the emotion such as anger, happiness, sad, surprise	Live video-based facial expressions to detect emotions while doing lab work online	Currently, the implemented approach works on identifying the emotions, but the further usage of identified emotions is missing	N/A
[6]	Have developed a framework to detect the eye-and-head movement to find out the concentration levels and sentiment during ongoing teaching sessions	Live video-based eye movements and head movement detection during an ongoing lecture	Currently, the implemented approach works on identifying the emotions, but the further usage of identified emotions is missing	87%
[7]	Have captured the audio, then applied sentence-level segmentation and have used WEKA classifier for emotion classification	The audio file is analyzed to find out the learning sentiment	The accuracy of classification can be improved further with different classifiers	96%
[8]	Have used webcam and microphone input and then used data fusion techniques to find out the learning emotions	Live audio and video to determine the learner’s mood in an ongoing class	The classification outcome is given to the user, not to the teacher	98.6%
[9]	A deep-learning algorithm CNN is used to detect the emotion of learning in online tutoring. They have used CK+, JAFFE, and NVIE datasets to train and test the models	Image-based facial expression recognition is performed to detect learning emotions	It was conducted as a simulation study; however, the actual testing in real time was not performed	N/A
[10]	Developed an emotion detection framework using the HAAR cascades method to analyze the eyes and mouth section from the images to detect emotion	Image-based facial expression recognition is performed to detect learning emotions	The outcome is drawn only based on two facial features, i.e., eyes and mouth	78–95%
[11]	Have used a support vector machine and random forest classifier to detect the emotion via the audio input	Live audio input is analyzed to find out the learning emotions	It lacked clear discrimination between features such as anger and happiness	80–93%

**Table 2:** Comparison of Kids’ Dataset.

Dataset	Sample	Subjects	Recording Condition	Elicitation Method	Expressions
LIRIS	208 video samples	12	Lab and home	Spontaneous	Disgust, happy, sad, surprise, neutral, fear
DEFSS	404 images	116	Lab	Posed	Happy, angry, fear, sad, neutral
NIMH-Chefs	480 images	59	Lab	Posed	Fear, happy, sad, neutral
DDCF	3200 images	80	Lab	Posed	Angry, sad, disgust, afraid, happy, surprised, contempt, neutral
CAFE	1192 images	90 women 64 men	Lab	Posed (exception: surprise)	Sad, happy, surprise, anger, disgust, fear, neutral
EmoReact	1102 videos	32 women 31 men	Web	Spontaneous	Happy, sad, surprise, fear, disgust, anger, neutral, valence, curiosity, uncertainty, excitement, attentiveness, exploration, confusion, anxiety, embarrassment, frustration
Authors’ dataset	53 videos, 8 lectures	4 men 4 women	Lab and home	Spontaneous + posed	Angry, sad, disgust, fear, happy, surprised, neutral

**Table 3:** Comparison of LIRIS and authors’ dataset accuracy against standard FER benchmark.

Model	LIRIS %	Our Dataset %	CK+/FER2013 %
InceptionV3	0.8406	0.8861	0.7404
InceptionResNet V2	0.8405	0.8854	0.7404
ResNet50 V2	0.8671	0.8998	0.7204
ResNet152 V2	0.8931	0.9098	0.7600
DenseNet121	0.8303	0.8907	0.5206/0.5144
DenseNet201	0.8307	0.8887	0.6475
VGG19	0.8100	0.8457	0.7270/0.8990

**Table 4:** Comparison of Model wise metrics on LIRIS.

Model on LIRIS	Accuracy %	Precision	Recall	F1 Score	Cohen’s Kappa
InceptionV3	0.8406	0.8418	0.8468	0.8458	0.8221
InceptionResNet V2	0.8405	0.8467	0.8448	0.8440	0.8211
ResNet50 V2	0.8671	0.8611	0.8649	0.8615	0.8454
ResNet152 V2	0.8931	0.8961	0.8994	0.8986	0.8665
DenseNet121	0.8303	0.8314	0.8367	0.8342	0.8136
DenseNet201	0.8307	0.8315	0.8368	0.8327	0.8137
VGG19	0.8100	0.8055	0.8034	0.8066	0.7898

**Table 5:** Comparison of Model wise metrics on Authors’ Dataset.

Model on LIRIS	Accuracy %	Precision	Recall	F1 Score	Cohen’s Kappa
InceptionV3	0.8861	0.8854	0.8860	0.8858	0.8864
InceptionResNet V2	0.8854	0.8867	0.8848	0.8852	0.8842
ResNet50 V2	0.8998	0.8997	0.8998	0.8997	0.8997
ResNet152 V2	0.9098	0.9061	0.9094	0.9086	0.9065
DenseNet121	0.8907	0.8905	0.8908	0.8909	0.8908
DenseNet201	0.8887	0.8884	0.8886	0.8888	0.8885
VGG19	0.8457	0.8455	0.8447	0.8455	0.8431

**Table 6:** CNN models’ detail.

Layers	Detail Layers	Filters	Units	Kernel Size	Stride	Activation
1	Input data	-	-	-	-	-
2	Pretrained ImageNet CNN	-	-	-	-	-
3	Dense_1	-	128	-	-	ReLu
4	Dense_2	-	128	-	-	ReLu
5	Dense_3	-	6 (if LIRIS) 7 (If authors dataset)	-	-	Softmax

**Table 7:** Final hyperparameters of experimental models.

CNN Used	Epoch Used in LIRIS	Batch Size Used in LIRIS	Epoch Used in Authors Dataset	Batch Size used in Authors Dataset	Pretrained ImageNet Weights Used	Additional Weight is Used
InceptionV3	30	32	6	16	✓	✓
InceptionResNet V2	30	32	6	16	✓	✓
ResNet50 V2	30	32	6	16	✓	✓
ResNet152 V2	30	32	6	16	✓	✓
DenseNet121	30	32	6	16	✓	✓
DenseNet201	30	32	6	16	✓	✓
VGG19	30	32	6	16	✓	✓

**Table 8:** Overall comparison of accuracy achieved on all kinds of dataset versions.

Model	WITHOUT FACE MESH %		WITH FACE MESH %	
	LIRIS	AUTHOR’S DATASET	LIRIS	AUTHOR’S DATASET
InceptionV3	0.8406	0.8861	0.8164	0.8661
InceptionResNetV2	0.8405	0.8854	0.8182	0.8626
ResNet50 V2	0.8671	0.8998	0.8356	0.8988
ResNet152 V2	0.8931	0.9098	0.8386	0.9087
DenseNet121	0.8303	0.8907	0.8157	0.8878
DenseNet201	0.8307	0.8887	0.8122	0.8821
VGG19	0.81	0.8457	0.8002	0.8236

## Data Availability

Data are available with the authors.
